# Supplementary material for: Multiple Antitumor Molecular Mechanisms Are Activated by a Fully Synthetic and Stabilized Pharmaceutical Product Delivering the Active Compound Sulforaphane (SFX-01) in Preclinical Model of Human Glioblastoma
Source: Pharmaceuticals (Basel). 2021 Oct 26;14(11):1082. doi: 10.3390/ph14111082 (PMC8626029; doi:10.3390/ph14111082)
Supplement: Supplementary file 1 [file pharmaceuticals-14-01082-s001.zip › pharmaceuticals-1333381-SI.pdf]

pharmaceuticals

MDPI

## Supplementary Figures

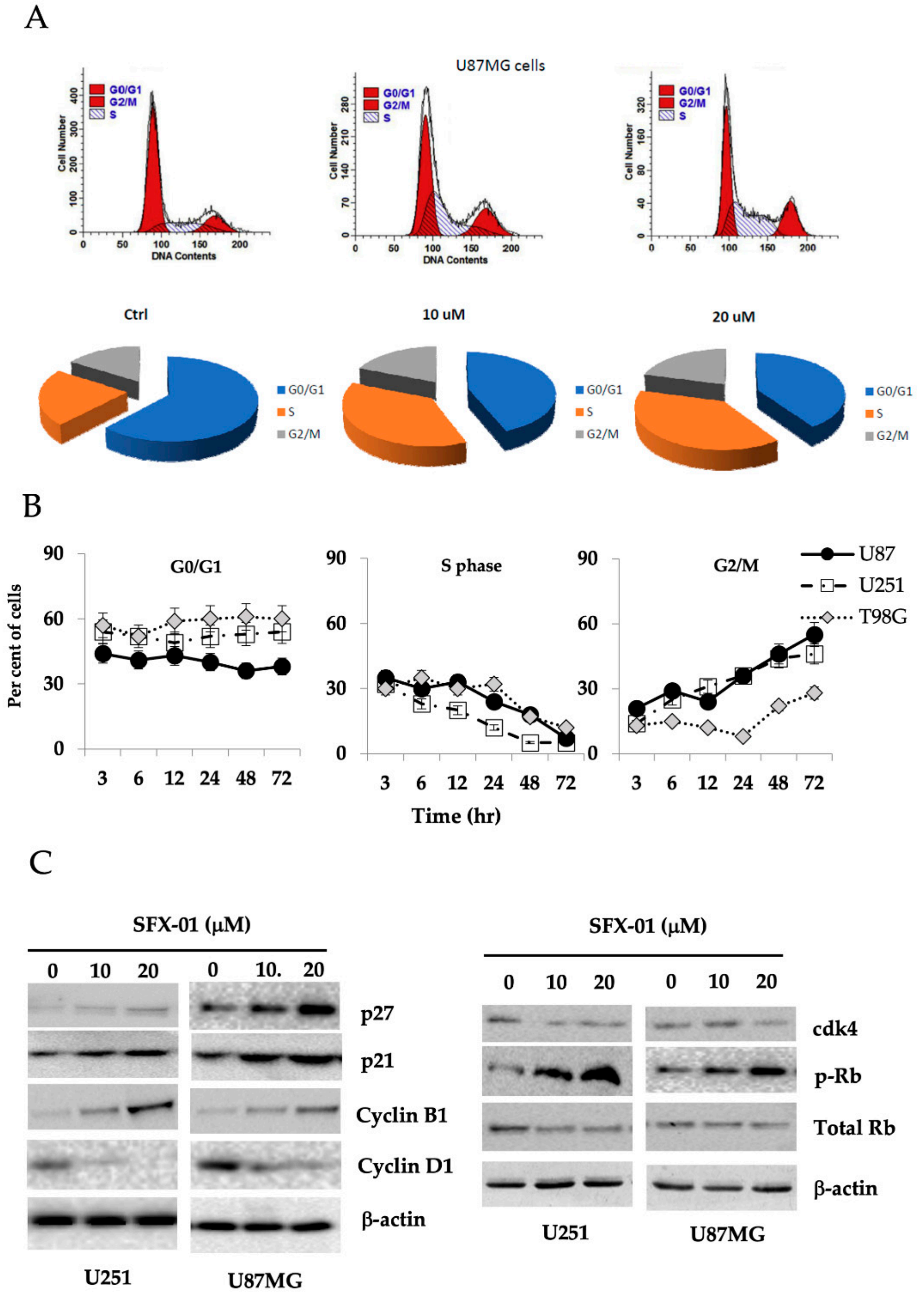


**Figure S1. SFX-01inhibits cell proliferation arresting cells to G2/M cell cycle phase**. (A) Cell cycle profiles of U87MG cells treated or not with 10 M SFX-01. (B) graphical distribution of SFX-01 cultures GBM cells in the time. (C) western blotting analyses of proteins of cell cycle in U87MG and U251 cells treated with 5 and 10 M SFX-01 for 24 hrs.

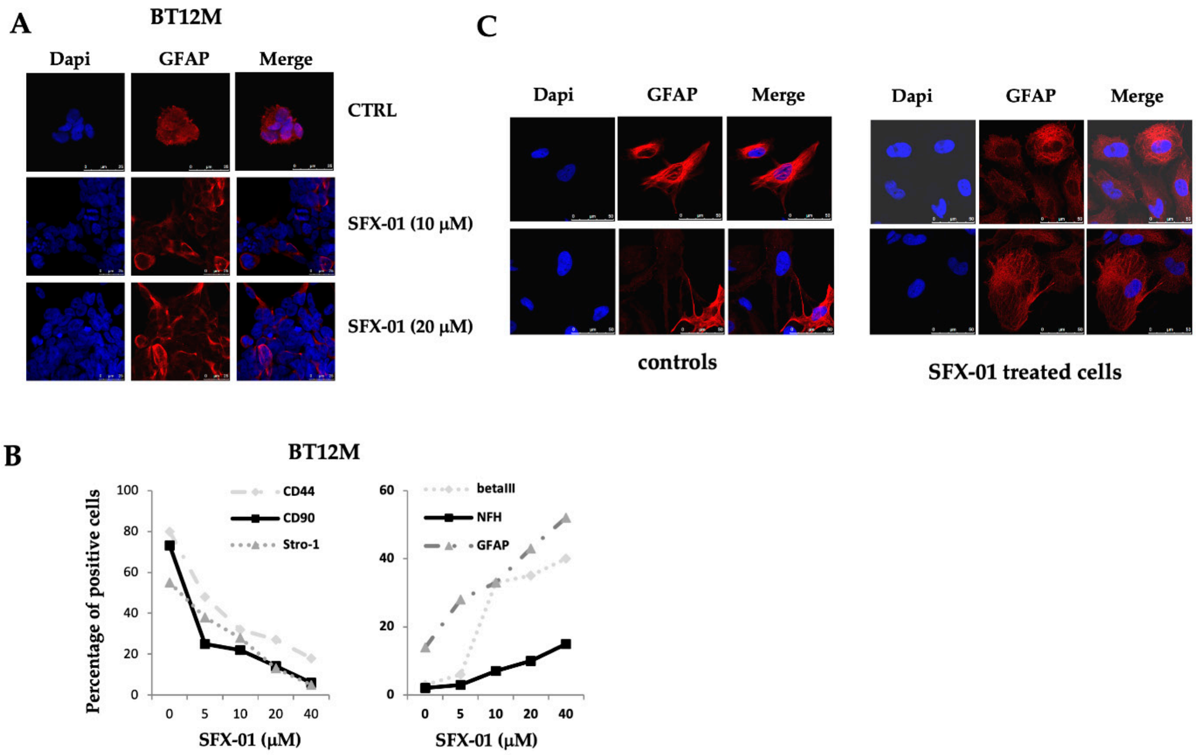


**Figure S2. SFX-101 modulates differentiation of glioma initiating cells.** (A) Expression of GFAP by confocal analyses in BT12M cells grown as neurospheres treated or not with 10 and 20 M SFX.01; (B) BT12M cells grown in adhesion and used to monitor the GFAP expression in control or 10 M treated SFX-01. (C) Percentage of expression of CD44, CD90, stro-1, beta-III, NFH and GFAP (graphical analyses performed in BT12M cells). (D) percentage of expression of CD44, CD90, Stro-1 b3-tubulin, GFAP and NFH by FACS analysis performed in BT48EF. BT50EF and GSCs-5.

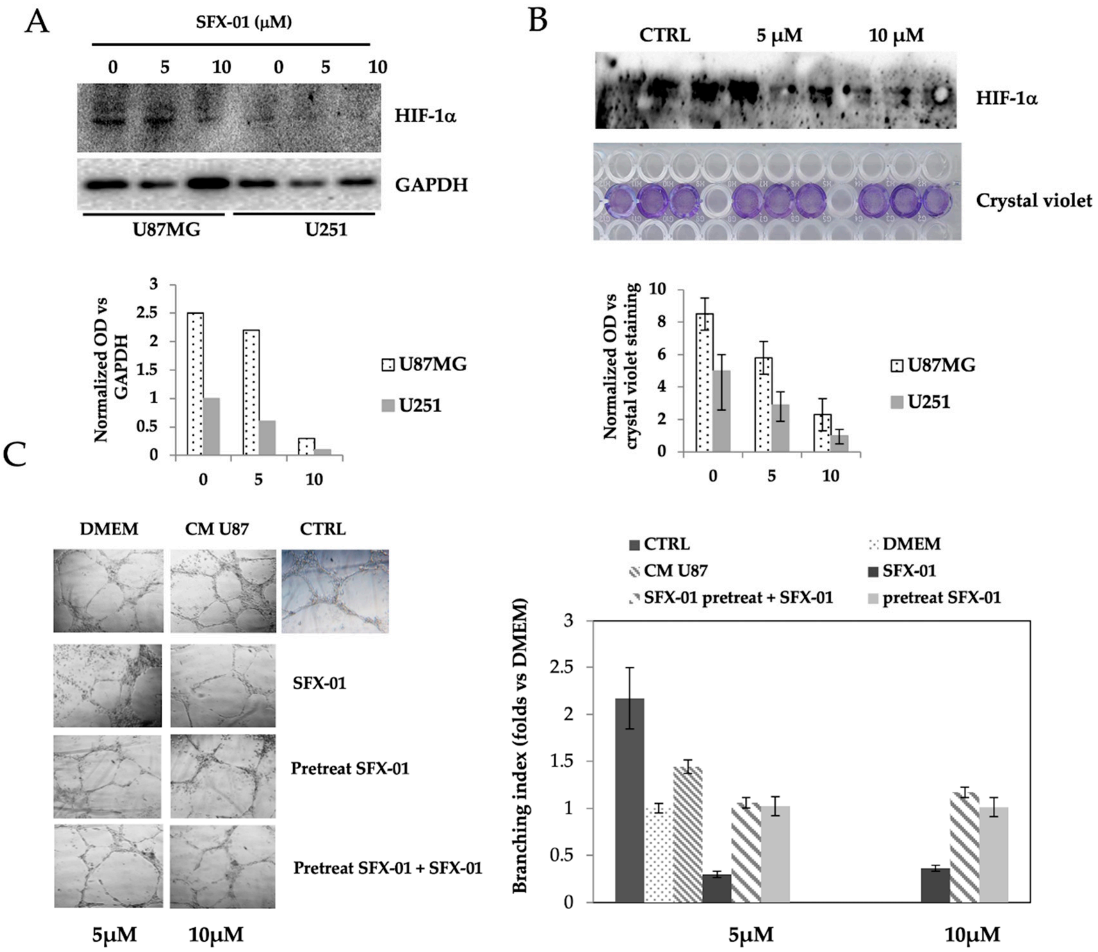


**Figure S3. SFX-101 modulated HIF1 expression and vasculomimicry**. (A) SFX-01 downregulates the expression of HIF-1$\alpha$ in cell extracts and western blot analyses; (B) the levels of HIF1 were also reduced in conditioned media from SFX-01 (5 and 10 mM) treated cells. Wester blots from Conditioned media (CMs) were analyzed in triplicate and values normalized for the amount of cells estimated from crystal violet stain. U87MG analyses were reported in WB whereas normalize values from U251 and U87MG were presented. (C) CM from U87MG were tested for the capacity to modulate tubule formation in endothelial angiogenic assay.

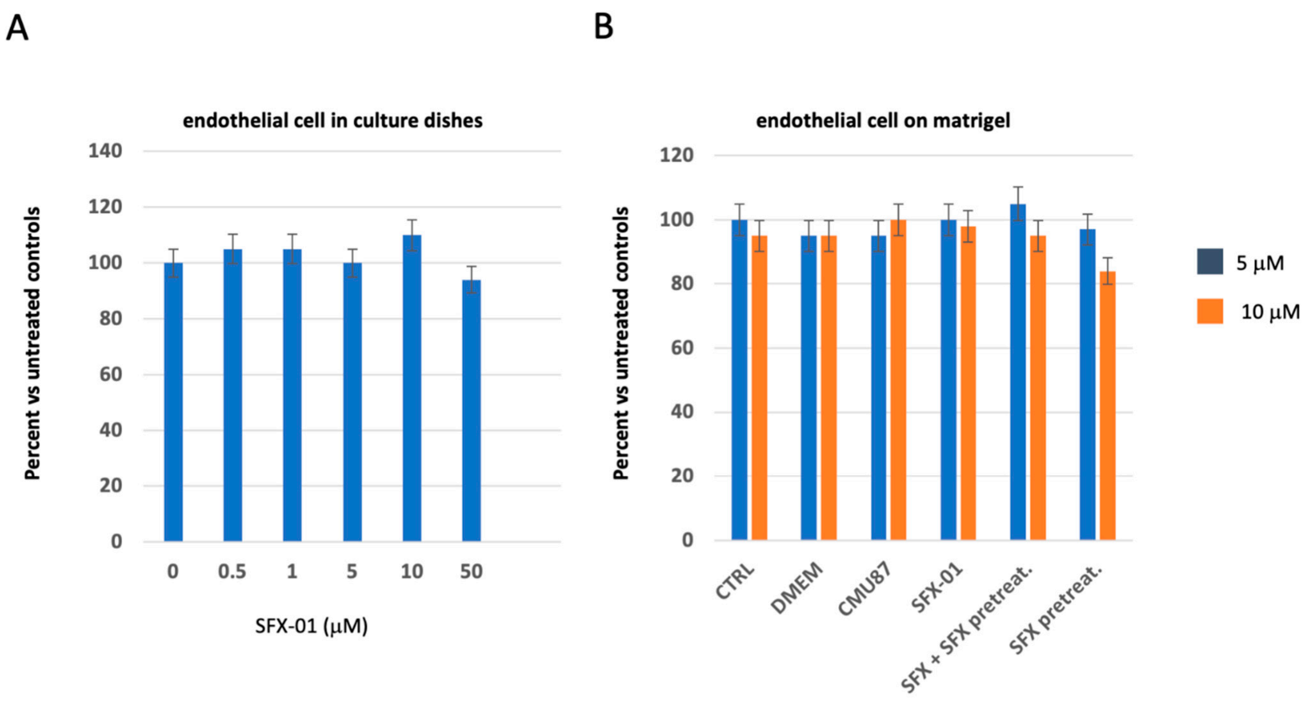


**Figure S4. SFX-101 is not effective in normal endothelial cells.** A) SFX-01 mediated DNA damage in a dose and time dependent manner. Comparison with Rt (4 Gy) used as positive control. The reduction of DNA damage observed in RT treated cells was not observed in SFX-01 treated cells suggesting a reduction of DNA repair (B) increased ROS levels are responsible for DNA damage. Effects of N-acetyl-L-cysteine (3 mM, NAC). (C) increased ROS levels are responsible for cytotoxic effects of SFX-01. Role of NAC.

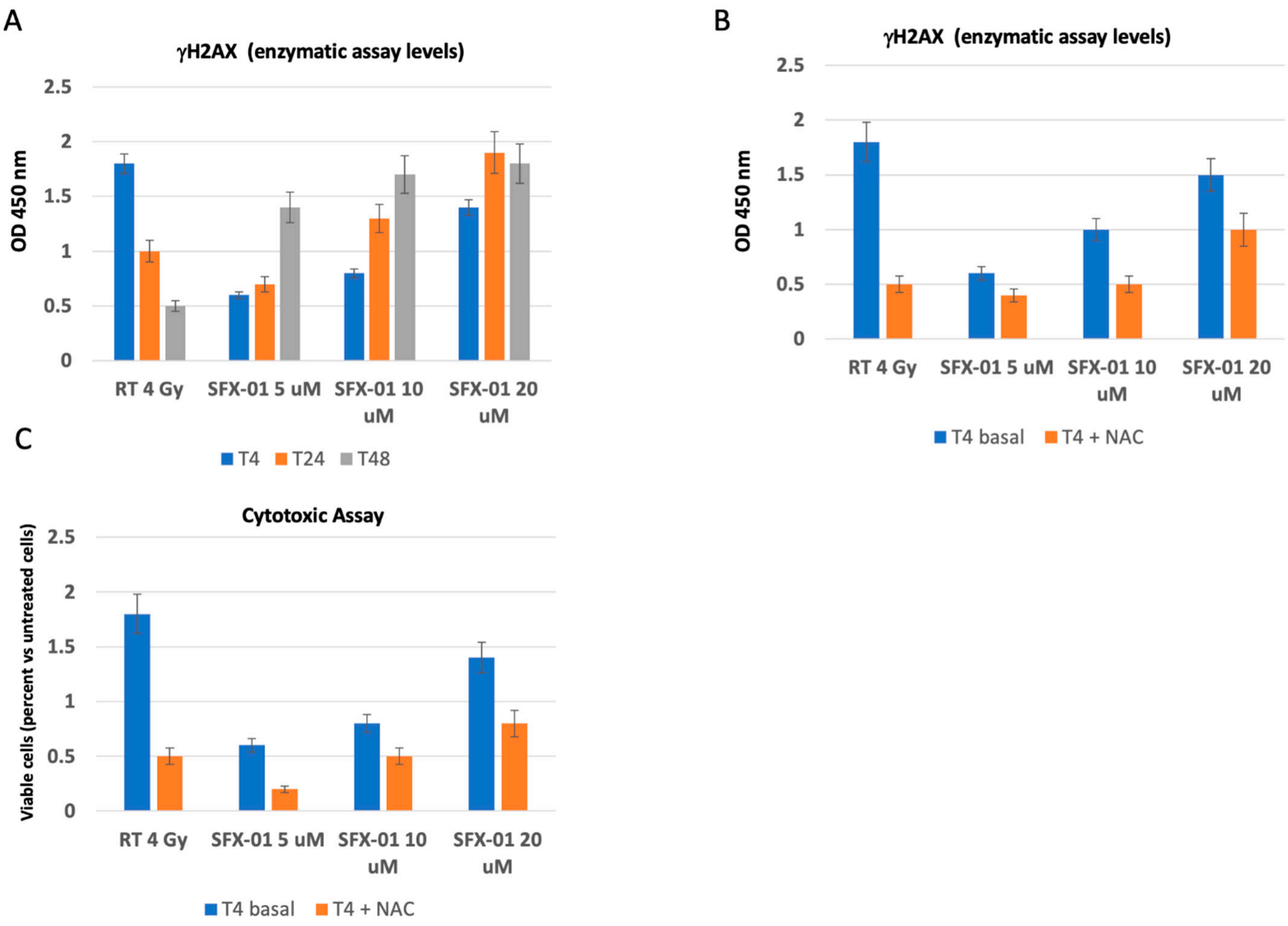


**Figure S5. SFX-101 modulated DNA damage.** ELISA determinations performed on nuclear extracts from untreated and treated cells con-firmed that SFX-01 mediated DNA damage and reduced DNA repair since γH2Ax levels did not com-pletely return to baseline in the SFX-01-treated cells with the respect to un-treated cells (supplementary data, figure S5A) In addition, the co-administration with the ROS-scavenger/e ROS inhibitor, N-acetyl-L-cysteine (supplementary data, figure S5B, C) reduced the gH2Ax levels and cell death in U87MG and U251 glioma cells indicating that ROS production is responsible for the DNA breaks and cell death in SFX-01 treated glioma cells.

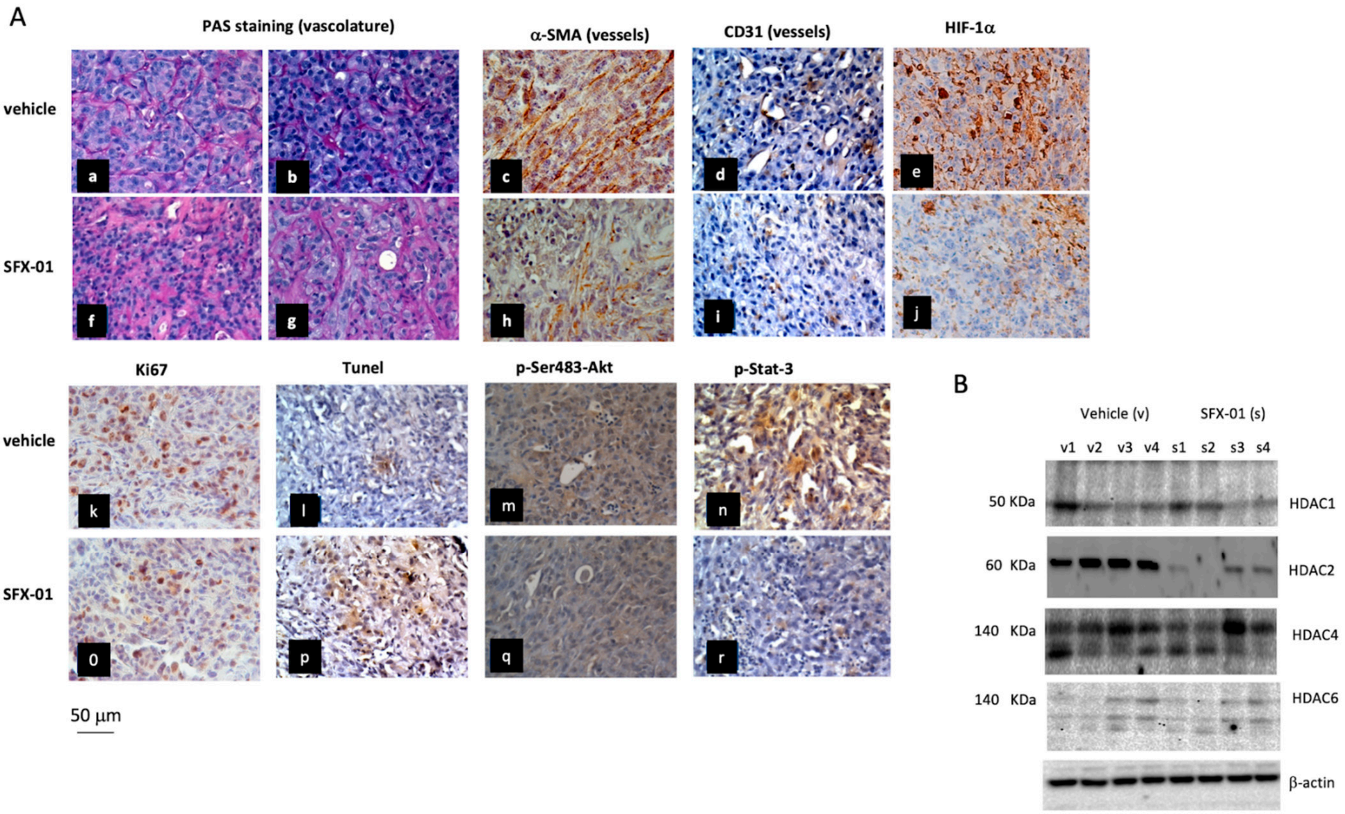


**Figure S6. Histological and immunohistochemical evaluations on SFX-101 treated tumors in vivo.** (A) representative images for PAS staining, Ki67, CD31 and HIF-1α Excised xenograft tissues from the GBM xenograft mice were examined for t expression of well-characterized tumor markers (Table II and supplementary data, Figure S6A). Ki67 was significantly reduced following the administration of SFX-01, suggesting a strong and significant inhibition of tumor cell proliferation and growth. TUNEL, an established marker of apoptotic DNA fragmentation, was elevated in tumor tissues, showing a dose-dependent effect of increased apoptosis. The decline in vessel counts (CD31-positive vessels and PAS-stained vascular mimicry-derived tubes) demonstrated decreased mi-crovasculature after SFX-01 administration. As sulforaphane shows inhibitory effects on HDAC, five tissues for treatment were also subjected to enzymatic and western blot analyses for the detection of HDAC activity and HDAC isoenzyme levels. HDAC activity and levels of HDAC1, HDAC2, HDAC4, and HDAC6 were reduced by SFX-01 administration.

**Table S1.** Time to Progression (TTP) statistical analyses for U87MG and T98G xenografts.

| | | | U87MG | |
|---|---|---|---|---|
| Groups | | TTP (Days) | Statistics | |
| 1. | CTRL | 13.7 ± 1.8 | | |
| 2. | SFX-01 | 21.6 ± 1.0 | CTRL vs SFX-01 | P<0.001 |
| 3. | RT | 15.9 ± 1.4 | CTRL vs RT<br>RT vs SFX-01 | Not significant<br>P<0.001 |
| 4. | SFX-01 + RT | 29.2 ± 1.2 | CTRL vs SFX-01 + RT<br>SFX-01 + RT vs RT<br>SFX-01 + RT vs SFX-01 | P<0.0001<br>P<0.0001<br>p<0.001 |
| | | T98G | | |
| 5. | CTRL | 13.2 ± 0.7 | | |
| 6. | SFX-01 | 17.0 ± 0.5 | CTRL vs SFX-01 P<0.001 | |
| 7. | RT | 15.0 ± 0.5 | CTRL vs RT<br>RT vs SFX-01 | Not significant<br>P<0.05 |
| 8. | SFX-01 + RT | 20.4 ± 1.1 | CTRL vs SFX-01 + RT<br>SFX-01 + RT vs RT<br>SFX-01 + RT vs SFX-01 | P<0.0001<br>P<0.005<br>p<0.05 |
